# The Atlanta College of Physicians and Surgeons

**Published:** 1899-04

**Authors:** 


### THE ATLANTA COLLEGE OF PHYSICIANS AND SURGEONS.

The first annual commencement of the Medical and Pharmaceutical Departments of The Atlanta College of Physicians and Surgeons was held in the Grand Opera House on the evening of April 3d. This was the first graduating class from the new organization, which is the old Atlanta Medical and Southern Medical Colleges consolidated. The session has been one of great prosperity in every respect, and the future of this institution bids fair to make it one of the foremost in the South. The session was well marked by the excellent work done by the new Faculty of Professors, and by the excellent examinations shown by the students themselves. The orator of the occasion was Hon. N. J. Hammond, of this city, who is so well known for his excellent flow of eloquence. This speech will be published in the May issue of The Journal–Record. We clip from the *Constitution* its remarks upon the commencement exercises:

"The feature of the evening was the speech of Hon. N. J. Hammond, which was a masterful effort and greatly applauded at its conclusion. As orator of the evening, he discussed at length the profession of medicine from its beginning up to the present time, and throughout the discourse he held the interested attention of the entire audience.

"The stage was tastefully decorated with palms, and was occupied by the members of the Board of Trustees and Faculty, and graduates of the college. The men were graduated in sections, and as each student received his diploma he was applauded by friends in the audience.

"During the progress of the exercises an excellent musical program was rendered by the special orchestra, and the remarks of all the speakers were interesting and appropriate.

"The men were graduated in the cap and gown, and after the conferring of the degrees the entire class of both departments of the college occupied seats on the right of the stage and were addressed by Hon. N. J. Hammond.

"The report of the Faculty, which was read by the Dean, Dr. W. S. Kendrick, was an interesting document and showed that the college was in an excellent condition.

"After the oration, the prizes were delivered by Mr. J. Carroll Payne, who made a few appropriate remarks regarding the awarding of the honors. This year it had been necessary to award five prizes, as two members of the class had made such excellent marks as to share the first two honors.

"The following received prizes:

"First honor—G. Street and H. S. Hansell.

"Second honor—L. C. Fischer and S. M. Samuels.

"Third honor—A. L. Fowler.

"Honorable mention—C. Thompson, J. C. Dover, G. S. Clark, H. S. Henderson, and B. C. Williamson.

"The excellent records of H. S. Hansell and O. L. Holmes entitle them to become members of the Grady Hospital staff of physicians.

"The program as rendered, which was interspersed with music, was as follows:

"Prayer—Rev. Dr. Rice.

"Report of Faculty—By the Dean, Dr. W. S. Kendrick.

"Conferring degrees—President Board of Trustees.

"Orator—Hon. N. J. Hammond, LL.D.

"Delivery of Prizes—Hon. J. Carroll Payne.

"Benediction—Rev. Dr. Rice.

"The following were the graduates:

"Medical Department—W. H. Perkinson, S. T. James, J. H. Brooks, H. S. Henderson, W. E. Stone, T. R. Moye, G. S. Clark,

S. M. Samuels, J. V. Davis, L. M. Ellis, J. R. Boring, W. M. White, H. L. Long, O. Daniel, A. E. Wheeler, L. C. Ward, A. L. Fowler, B. C. Wilson, J. S. Alsbrook, H. E. Center, B. H. Maynard, H. R. Moore, T. G. Cunningham, G. D. McClain, J. C. Oover, H. R. Donaldson, T. Toepel, W. R. Reed, E. H. Harris, J. L. Beam, C. Thompson, W. B. Emory, W. Cawhern, R. Comer, O. Lyndon, O. C. West, V. A. Peterson, L. C. Fischer, H. S. Hansell, J. W. E. H. Beck, J. M. McDonald, J. G. Smith, L. E. Powell, W. H. DeLacy, J. W. Keir, T. K. Slaughter, J. B. Dalton, M. L. Stoegh, S. H. Smith, O. L. Holmes, G. Street, W. S. Ansley, R. B. Patterson, T. H. Thrasher.

"Pharmaceutical Department—L. L. Scarborough, Choccolocco, Ala.; R. B. Beadles, Atlanta, Ga.; J. M. Cleveland, Elberton, Ga.; T. A. Duke, Douglasville, Ga.; C. G. Durham, Acworth, Ga.; W. W. Fincher, Waleska, Ga.; J. W. Gallaway, Monroe, Ga.; W. H. Holbrook, M.D., Decora, Ga.; L. J. LeSueur, Roberta, Ga.; J. T. Lightner, Schley, Ga.; W. R. Moore, Sharon, Ga.; J. E. Pittman, Athens, Ga.

"No prizes were given in the Pharmaceutical Department, but L. L. Scarborough received the highest mark for general proficiency and T. P. Bell came second. The difference between the two was only one twenty-fifth of one per cent."

